# High‐Throughput Immunoassays for Cavin‐4 IgG: A Diagnostic Tool for Immune‐Mediated Rippling Muscle Disease

**DOI:** 10.1002/acn3.70012

**Published:** 2025-02-17

**Authors:** Reghann G. LaFrance‐Corey, Haidara Kherbek, Nimalan Harinesan, Margherita Milone, Naveen K. Paramasivan, Pallab Sarker, Andrew M. Knight, Carley Karsten, Surendra Dasari, Teerin Liewluck, William J. Litchy, Sean J. Pittock, John R. Mills, Divyanshu Dubey

**Affiliations:** ^1^ Department of Laboratory Medicine and Pathology Mayo Clinic Rochester Minnesota USA; ^2^ Department of Neurology Mayo Clinic Rochester Minnesota USA; ^3^ Center for Multiple Sclerosis and Autoimmune Neurology Mayo Clinic Rochester Minnesota USA

**Keywords:** autoimmune myopathy, caveolae‐associated protein 4 (cavin‐4), enzyme‐linked immunosorbent assay (ELISA), immune‐mediated rippling muscle disease (iRMD), phage immunoprecipitation sequencing (PhIP‐Seq)

## Abstract

Cavin‐4 was identified as a potential autoantigen for immune‐mediated rippling muscle disease (iRMD). To validate this, we developed and tested various immunoassays, including a cell‐based assay (CBA), cavin‐4 recombinant protein ELISA, and multi‐peptide ELISA. Among 19 iRMD patients, all exhibited muscle rippling, and 13 had percussion‐induced mounding. All immunoassays demonstrated clinical and analytical specificities greater than 95%. The protein ELISA had the highest sensitivity (94.7%) and specificity (99.9%), outperforming CBA (sensitivity 89.5%, specificity 99.6%) and the multi‐peptide ELISA (sensitivity 79.0%, specificity 97.2%). Our results suggest that the cavin‐4 protein ELISA is a promising tool for high‐throughput clinical testing in iRMD.

## 1 Introduction

Immune‐mediated rippling muscle disease (iRMD) is an autoimmune myopathy characterized by wavelike muscle contractions (rippling) and percussion‐ or stretch‐induced muscle mounding [1, 2]. Previously, we identified caveolae‐associated protein 4 (cavin‐4) autoantibodies in iRMD using phage immunoprecipitation sequencing (PhIP‐Seq) [1]. Another independent lab confirmed our findings and reported the first case of cavin‐4 IgG seropositive iRMD associated with thymoma [3]. This study aims to report additional cases of cavin‐4 IgG‐positive iRMD and identify a high‐throughput assay suitable for clinical laboratory testing to aid in the diagnosis of iRMD.

## 2 Methods

The Mayo Clinic Institutional Review Board approved the study (protocols 16‐009814 and 18‐010637). All patients at the Mayo Clinic whose medical records were reviewed provided written consent for medical research.

### 2.1 Patient Selection

Between 2000 and 2024, we collected 10 archived and 9 additional patient sera (19 sera total) that were clinically diagnosed with iRMD [1, 2]. Twelve of these cases had undergone muscle biopsy that demonstrated a mosaic pattern for caveolin‐3 staining [1]. Serum samples from disease controls included patients with hypergammaglobulinemia, antinuclear antibody, antimitochondrial antibody, multiple sclerosis, inclusion body myositis, myositis‐related syndromes, and interstitial lung disease, amyotrophic lateral sclerosis, autoimmune encephalitis, necrotizing autoimmune myopathies, and peripheral nerve hyperexcitability syndromes (Table S1). Healthy controls were also tested on each platform.

### 2.2 Phage Immunoprecipitation Sequencing

Sera from 14 patients with clinical characteristics of iRMD were evaluated by whole–human proteome phage immunoprecipitation sequencing (PhIP‐Seq) as previously described [1]. Enrichment scores > 10 were considered to indicate the presence of autoantibodies. An epitope map generated by iRMD patient sera run on PhIP‐Seq was used to identify the peptides of interest for multi‐peptide enzyme‐linked immunosorbent assay (ELISA).

### 2.3 Cavin‐4 Peptide Synthesis

Peptide AA sequences cavin‐4.1 (DEDQDAALTI VTVLDKVASI VDSVQASQKR IEERHREMEN AIKSVQIDLL KLSQSHSNT GHIINKLFEK TRKV), cavin‐4.2 (TENQEEDDDD IFDPPVDLSS DEEYYVEESR SARLRKSGKE HIDNIKKAFSK ENMQKTRQNL DKKVNRIRTR IVTPERRERL RQSGERL), and cavin‐4.3 (CAREMGVDII ARSESLGPIS ELYSDELSEP EHEAARPVYP PHEGREIPTP) were synthesized with an additional N‐terminal cysteine residue by the Mayo Clinic Peptide Synthesis Core. Peptides were reconstituted at 1 mg/mL in phosphate‐buffered saline (PBS) and stored at −80°C.

### 2.4 Immunoassays

Detailed methodological procedures for the cavin‐4 peptide ELISA, protein ELISA, and cell‐based assay (CBA), including optimization of peptide and protein concentrations, sample and secondary antibody dilutions, and experimental conditions, are provided in the Supporting Information.

### 2.5 Statistical Analysis

Sensitivity, specificity, and accuracy were calculated using the Clopper‐Pearson exact method. Correlation analysis of clinical data was performed using Spearman correlations. Receiver‐operating curve (ROC) analyses were used to evaluate ELISA OD cut‐offs. All analyses were performed using IBM SPSS version 28.

## 3 Results

### 3.1 iRMD Patients

**FIGURE 1:**
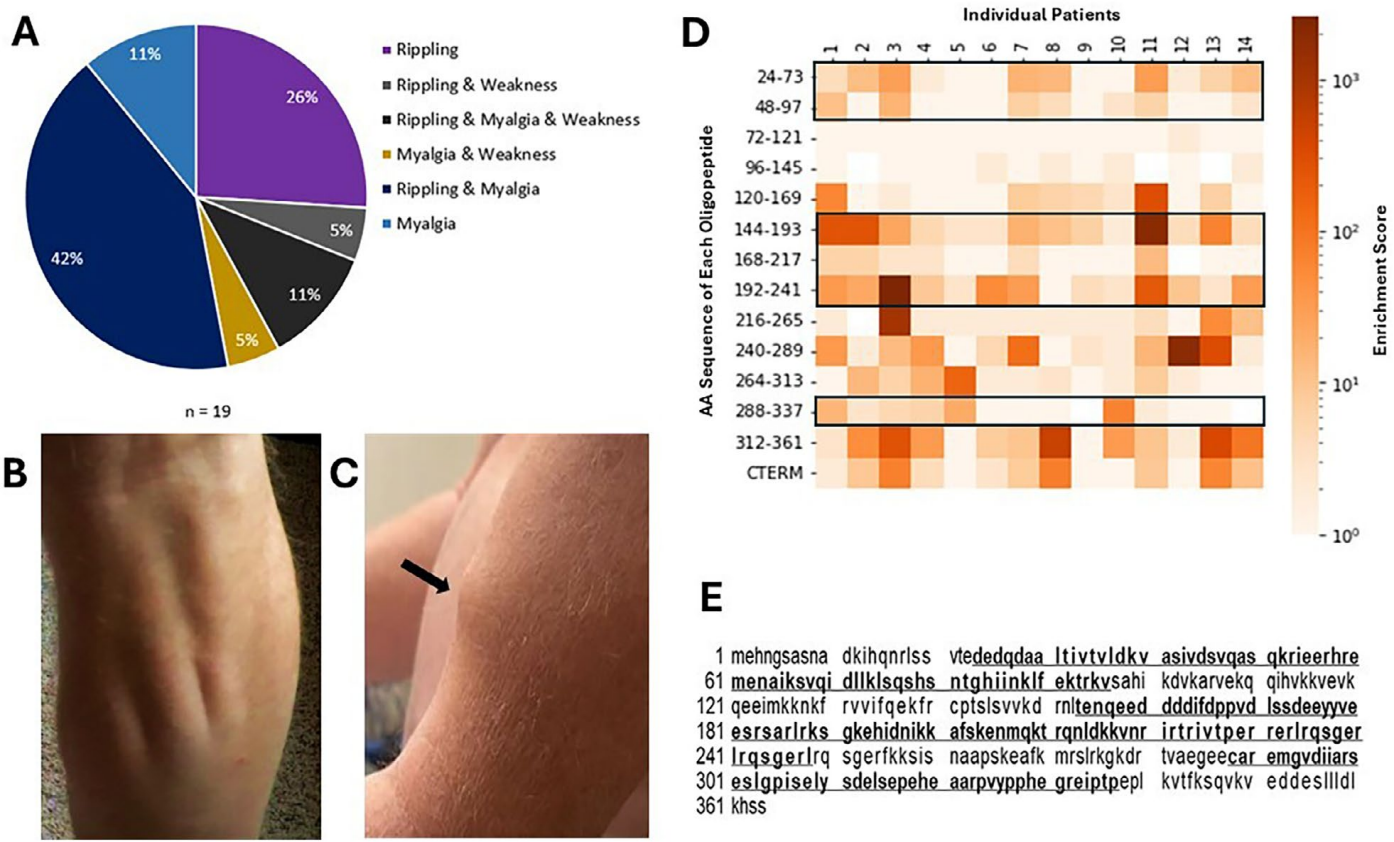
Clinical presentation of immune‐mediated rippling muscle disease (iRMD) and identification of cavin‐4 autoantibodies in iRMD patient sera using phage immunoprecipitation sequencing (PhIP‐Seq). (A) Distribution of presenting symptoms in patients with iRMD. (B) Rippling of calf muscle in a patient with iRMD on getting up from his chair. (C) Muscle mounding (black arrow) noted in the arm of the same patient following percussion with his fingers. (D) Heatmap indicating the PhiP‐Seq enrichment scores of iRMD patient serum. Black boxes indicate oligopeptide sequences included in the multi‐peptide ELISA. (E) Peptide sequence of fragments 1, 2, and 3 are bold and underlined.

The median age of presentation of our cohort of iRMD patients was 61 years (range: 26–79) with 13/19 being male. The most common presenting symptoms included rippling muscles (*n* = 13), muscle twitching (*n* = 5), and myalgias (*n* = 7) (Figure 1A). Co‐existing autoimmune diseases were noted in 5/19 patients (Type 1 diabetes, *n* = 1; Hashimoto's thyroid disease, *n* = 3; pernicious anemia, *n* = 1; myasthenia gravis, *n* = 3). Clinical examination demonstrated the presence of rippling muscles (Figure 1B; Video S1) in all patients and muscle mounding (Figure 1C; Video S2) with percussion in 13/19 patients. Serum creatine kinase was elevated in 15/17 patients (median 423 U/L; range 132–885 U/L) and co‐existing antibodies were noted in 8/18 patients (striational muscle IgG, *n* = 7; anti‐acetylcholine receptor IgG [AChR IgG], *n* = 8 [median titer 3.82 nmol/L, range 0.12 nmol/L—6.31 nmol/L]). Twelve patients were treated with immunotherapy, with four having symptom resolution, one having partial improvement, five having stabilization of symptoms, and two patients with progression of symptoms.

### 3.2 ELISA Development

We screened sera from 14 iRMD patients by PhIP‐seq and identified several common peptide hits corresponding to different regions of the cavin‐4 protein (Figure 1D). Peptide ELISA was developed using oligopeptide sequences identified by PhIP‐seq (Figure 1E). ROC analysis was performed using ELISA optical density (OD) values from healthy control sera (protein ELISA, *n* = 120; multi‐peptide ELISA, *n* = 47) and iRMD patient sera to determine the OD cutoff value of 1.0 (Figure S1).

### 3.3 Immunoassay Method Comparison and Performance

The performance of the evaluated IgG ELISAs and CBAs varied (Table S2). To assess the specificity of the putative autoantigen, serum samples from patients with autoimmune CNS diseases or immune‐mediated myopathies were screened by both ELISA methods and CBA.

**FIGURE 2:**
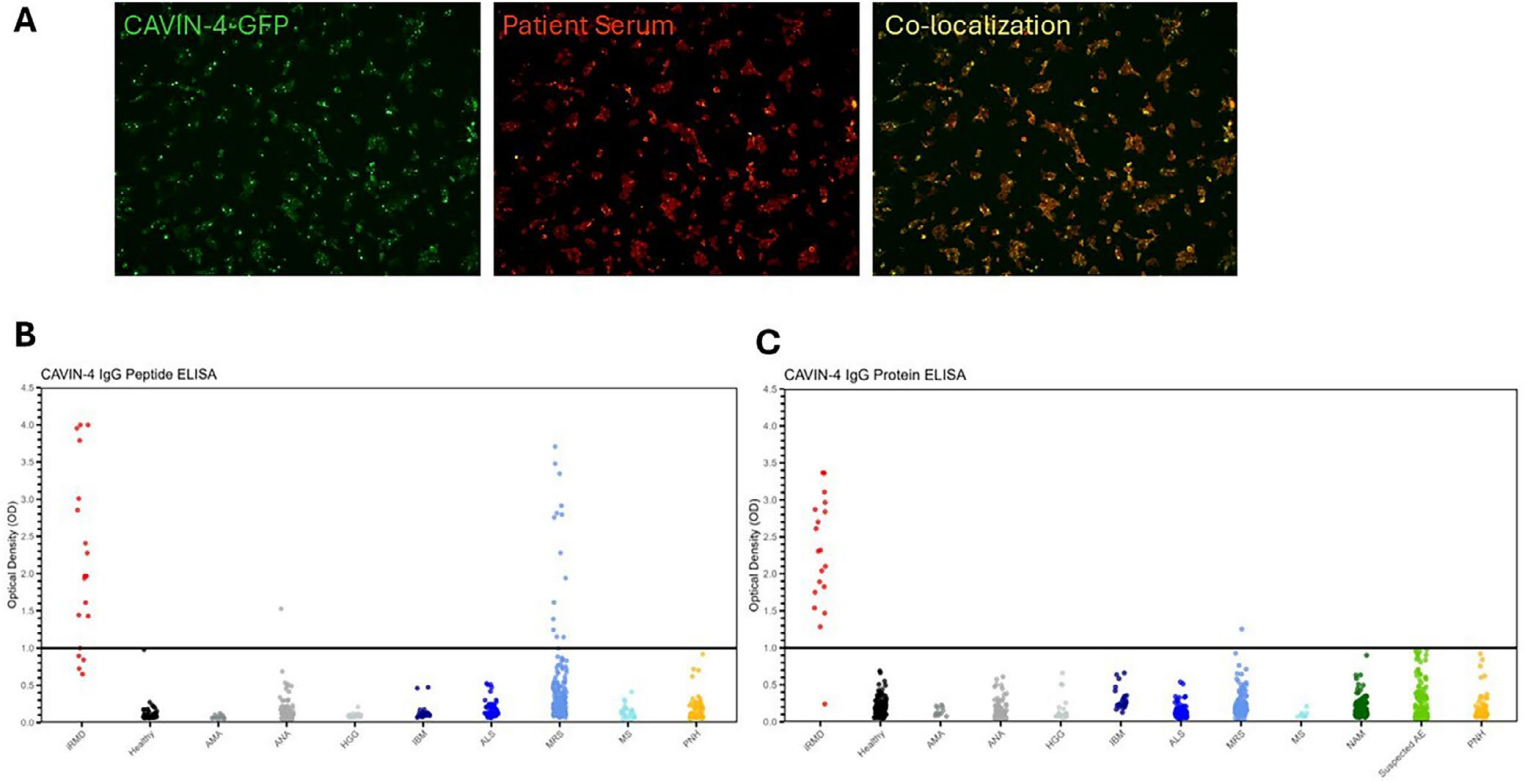
High throughput assays for detection of cavin‐4 IgG. (A) Cell‐based assay showed colocalization between cavin‐4‐GFP and serum from patients with immune‐mediated rippling muscle disease. (B) Specificity of the cavin‐4 multi‐peptide ELISA. (C) Specificity of the cavin‐4 protein ELISA. For (B) and (C), samples were considered positive if they had an optical density value ≥ 1.0 (solid line). AE, autoimmune encephalitis; ALS, amyotrophic lateral sclerosis; AMA, antimitochondrial antibody; ANA, antinuclear antibody; HGG, hypergammaglobulinemia; IBM, inclusion body myositis; MRS, myositis related syndrome; MS, multiple sclerosis; NAM, necrotizing autoimmune myopathies; PNH, peripheral nerve hyperexcitability.

We tested sera from 19 patients with iRMD for cavin‐4 IgG by CBA, using cavin‐4‐transfected HEK293 cells as substrate (Figure 2A). Of the 19 patients, 17 (89%) tested positive for cavin‐4 IgG. Two sera that were negative by CBA were screened by whole proteome PhIP‐seq and bound selectively to cavin‐4 peptides. We then performed ELISA screening and found that 15 patients (79%) were positive on the multi‐peptide ELISA (Figure 2B); 18 (95%) were positive on the protein ELISA (Figure 2C). The clinical information of the two patients who had discordant results is detailed in Table S3. None of the sera from 123 healthy control individuals were positive by CBA, and one disease control (141 tested) was false‐positive. Only one disease control out of 1407 tested was a false positive by cavin‐4 protein ELISA, while 15 out of 474 were false positives on the multi‐peptide ELISA. Some multi‐peptide ELISA false positives were assessed with PhIP‐Seq (Table S4), but no IgG binding to cavin‐4 oligopeptide was found, indicating that these patients with diverse rheumatologic diseases do not have IgG targeting the cavin‐4 protein.

Table 1 shows the comparative performance of each assay. Protein ELISA showed the highest sensitivity (94.7%), followed by CBA (89.5%) and multi‐peptide ELISA (79.0%). Protein ELISA also showed the highest specificity (99.9%), followed by CBA (99.6%) and multi‐peptide ELISA (97.2%). The same trend was observed for accuracy (99.9%, 98.9%, and 96.6% for protein ELISA, CBA, and multi‐peptide ELISA, respectively).

**TABLE 1:** Sensitivity, specificity, accuracy, and false positive rate of the three evaluated cavin‐4‐IgG immunoassays.

Cavin‐4 Protein ELISA			Cavin‐4 Peptide ELISA			Cavin‐4 CBA		
Statistic	Value	95% CI	Statistic	Value	95% CI	Statistic	Value	95% CI
Sensitivity	94.7%	74.0%–99.9%	Sensitivity	79.0%	54.4%–94.0%	Sensitivity	89.5%	66.9%–98.7%
Specificity	99.9%	99.6%–100.0%	Specificity	97.2%	95.4%–98.4%	Specificity	99.6%	97.8%–100.0%
Accuracy	99.9%	99.5%–100.0%	Accuracy	96.6%	94.7%–97.9%	Accuracy	98.9%	96.8%–99.8%
False positive (%)	1/1485 (0.07%)		False positive (%)	15/545 (2.8%)		False positive (%)	1/250 (0.4%)	
False negative (%)	1/19 (5.2%)		False negative (%)	4/19 (21.0%)		False negative (%)	2/19 (10.5%)	

Abbreviations: cavin‐4, caveolae‐associated protein 4; CBA, cell based assay; CI, confidence interval; ELISA, enzyme linked immunosorbent assay.

## 4 Discussion

The findings of this study expand on our previous research by documenting additional cases and further validating cavin‐4 IgG as a disease‐specific biomarker for iRMD. Utilizing various immunoassays such as CBA, protein ELISA, and multi‐peptide ELISA assays, we achieved specificity and diagnostic accuracy exceeding 95%. Notably, protein ELISA and CBA exhibited the highest specificity, with protein ELISA also demonstrating the greatest sensitivity and overall diagnostic accuracy among the three methods.

The limited specificity and sensitivity of the multi‐peptide ELISA may be due to cryptic or linear epitope presentation by short synthetic peptides [4, 5], and because cavin‐4 IgG binds to multiple different epitopes, some of which are not covered by this assay [4]. While the CBA produced only 1 false‐positive among 264 controls, its sensitivity in iRMD cases dropped to 89.3% due to two negative results. In contrast, the protein‐based ELISA outperformed both other assays in terms of accuracy, sensitivity, and specificity.

The ELISA's ability to test in a 96‐well format offers a scalable solution compared to the 8‐well chamber slides used in CBA. These results suggest that cavin‐4 protein ELISA is a practical and high‐throughput method, facilitating the clinical development of an assay for cavin‐4 IgG detection and allowing timely iRMD diagnosis.

This method not only aids in diagnosing iRMD but also in distinguishing it from neurological disorders with similar presentations, such as Isaac syndrome and cramp fasciculation syndrome [6, 7]. It may even reduce the need for invasive procedures like muscle biopsies. The protein ELISA achieved the necessary specificity to proceed with further clinical validation, and future studies should aim to standardize cavin‐4 IgG detection protocols across clinical laboratories.

Examining rare diseases like iRMD presents inherent challenges. A primary limitation of this study is the small sample size. Consequently, studies with larger sample sizes are required to identify significant differences between the assays mentioned. Several factors may influence the sensitivity of the CBA assay. The cavin‐4 antigen, fused with GFP at the N‐terminus, often localizes within the cytosol as aggregates. Additionally, a marginally higher background signal, potentially introduced by fixation and permeabilization in the cavin‐4 CBA, may confound the detection of low‐titer cases [8].

## 5 Conclusions

Cavin‐4 IgG serves as a reliable disease‐specific biomarker for iRMD. Our results suggest the cavin‐4 protein ELISA is a promising tool for high‐throughput clinical testing in iRMD. The presentation of muscle rippling and mounding, with or without co‐existing MG, should prompt further investigation for iRMD. Our findings actively drive the development of clinical tests for cavin‐4 IgG, facilitating its integration as a serological biomarker in the diagnosis and management of iRMD.

## Author Contributions

Conception and design of the study: D.D., R.G.L.‐C. Acquisition and analysis of data: All authors. Drafting the manuscript or figures: H.K., R.G.L.‐C., and D.D. Study supervision: D.D.

## Conflicts of Interest

R.G.L.‐C., H.K., N.H., N.K.P., P.S., C.K., S.D., W.L., J.R.M. report no conflicts of interest. D.D. has consulted for Immunovant and Astellas Pharmaceuticals, compensation for which is paid directly to Mayo Clinic; and has patents pending for Kelch‐like protein 11 IgG, Leucine Zipper 4 IgG, and Caveolae Associated Protein‐4 IgG as markers of neurological autoimmunity. A.M.K. has a patent pending for Caveolae‐Associated Protein‐4 IgG as a marker of immune‐mediated rippling muscle disease. T.L. has received research support from the neurology department at the Mayo Clinic. S.J.P. has patents for neuromyelitis optica autoantibodies as a marker for neoplasia issued and for methods for treating neuromyelitis optica by the administration of eculizumab to an individual who is aquaporin‐4‐IgG autoantibody positive; has a patent pending for GFAP, Septin 5, MAP1B, KLHL11, PDE10A, cavin‐4 IgGs as markers of neurological autoimmunity and paraneoplastic disorders; has consulted for Alexion, Euroimmune, Medimmune, Astellas, Genentech, Sage Therapeutics, and Prime Therapeutics; and has received research support from Grifols, Alexion, National Institutes of Health, Guthy Jackson Charitable Foundation, and Autoimmune Encephalitis Alliance; all compensation for consulting activities is paid directly to Mayo Clinic. M.M. has received research support from the neurology department at the Mayo Clinic and Center for Clinical and Translational Science, a care center grant award from the Muscular Dystrophy Association, and compensation to serve as associate editor of Neurology Genetics. M.M. has a patent pending for Caveolae Associated Protein‐4 IgG as a marker of immune‐mediated rippling muscle disease. No other disclosures were reported.

## Supporting information


Data S1.

Tables S1–S4.
**Figure S1**. Comparison of Peptide and Protein ELISA ROC and Area Under the Curve in iRMD Patients as Compared to Healthy Controls.

**Video S1.** Rippling of calf muscles in a patient on getting up from a chair.

**Video S2.** Muscle mounding of biceps post percussion with a finger.

## Data Availability

The data that support the findings of this study are available from the corresponding author upon reasonable request.
