# Supplementary material for: High‐Throughput Immunoassays for Cavin‐4 IgG: A Diagnostic Tool for Immune‐Mediated Rippling Muscle Disease
Source: Ann Clin Transl Neurol. 2025 Feb 17;12(4):876–80. doi: 10.1002/acn3.70012 (PMC12040518; doi:10.1002/acn3.70012)
Supplement: Supplementary file 1 — Data S1. [file ACN3-12-876-s004.docx]

**Supplementary Methods**

***Protein- or peptide-based ELISA***

ELISAs were performed in 96-well Immulon 2HB plates coated with either recombinant human MURC GST (N-Term) protein (15 ng/well; Novus cat #H00347273) or cavin-4 peptides (20 ng per peptide per well) diluted in 0.01M NaPO_4_ (pH 7.4). Coated plates were incubated for 2 hours at 37 °C or overnight at 4 °C, then washed three times with PBS containing 0.05% Tween-20. Plates were then blocked for 1 hour at 37 °C with PBS containing 0.05% Tween-20 and 10% goat serum. Sera were screened in duplicate at 1:200 dilution in PBS containing 0.05% Tween-20 and 10% goat serum. After incubating for 90 minutes at 37°C, and washing 6 times, a secondary antibody was added and incubated at 37 °C for 1 hour (horseradish peroxidase–labeled goat antihuman IgG H+L diluted 1:30,000 in PBS containing 0.05% Tween-20 and 10% goat serum). After 6 washes, the enzyme substrate TMB was added and incubated for 20 min at 37 °C; the colorimetric reaction was stopped by adding 1M NaOH. The plate was read at an absorbance of 450 nm with an Agilent microplate reader using GEN5 software. IgGs yielding an optical density (OD) greater than 1.0 were considered positive. The positive samples were titrated at doubling dilutions from 1:50 to 1:6400.

***Cell-based assay***

HEK293 cells were transfected with full-length green fluorescent protein (GFP)-tagged human cavin-4 expressing plasmid (CAVIN1_OHu23701C_pcDNA3.1(+)-C-eGFP, Genscript). Post-transfection HEK293 cells were incubated for 16–24 hours at 37 °C in a humidified atmosphere of 95% air + 5% CO_2_. Cells were then fixed (4% paraformaldehyde in PBS, 15 minutes), permeabilized (0.2% Triton X-100 in PBS, 10 minutes), and blocked (10% normal goat serum in PBS, 30 minutes). After washing with PBS, cells were incubated for 60 minutes with patient serum diluted 1:200 in 10% normal goat serum in PBS. After washing with PBS, cells were incubated for 45 minutes with secondary antibodies (1:200 tetramethylrhodamine [TRITC]-conjugated goat anti-rabbit IgG and goat anti-human IgG; Southern Biotech, Birmingham, AL). Coverslips were mounted using ProLong Gold antifade medium containing 4,6- diamidino-2-phenylindole (DAPI) (Molecular Probes, Thermo Fisher Scientific). Assays were scored by at least two independent reviewers.
