# Supplementary material for: High‐Throughput Immunoassays for Cavin‐4 IgG: A Diagnostic Tool for Immune‐Mediated Rippling Muscle Disease
Source: Ann Clin Transl Neurol. 2025 Feb 17;12(4):876–80. doi: 10.1002/acn3.70012 (PMC12040518; doi:10.1002/acn3.70012)
Supplement: Supplementary file 2 — Tables S1–S4. Figure S1. Comparison of Peptide and Protein ELISA ROC and Area Under the Curve in iRMD Patients as Compared to Healthy Controls. [file ACN3-12-876-s003.docx]

**Supplementary Table 1:** Summary of methods by which autoantibodies to cavin-4 were identified in immune-mediated rippling muscle disease.

| **Patient number** | **Maximum PhIP-Seq enrichment score** | **CBA** | **Protein ELISA** | **Protein ELISA titers** | **Peptide ELISA** |
| --- | --- | --- | --- | --- | --- |
| 1 | 268 | pos | pos | 1600 | pos |
| 2 | 260 | pos | pos | 12800 | pos |
| 3 | 2616 | pos | pos | 3200 | pos |
| 4 | 35 | pos | pos | 400 | pos |
| 5 | 151 | pos | pos | 400 | pos |
| 6 | 53 | pos | pos | 1600 | pos |
| 7 | 114 | pos | pos | 800 | neg |
| 8 | 500 | pos | pos | 6400 | pos |
| 9 | 7 | neg | neg | <200 | pos |
| 10 | 67 | neg | pos | 400 | neg |
| 11 | 1968 | pos | pos | 1600 | pos |
| 12 | 1830 | pos | pos | 3200 | neg |
| 13 | 356 | pos | pos | 6400 | pos |
| 14 | 92 | pos | pos | 1600 | pos |
| 15 | NA | pos | pos | 3200 | pos |
| 16 | NA | pos | pos | 12800 | pos |
| 17 | NA | pos | pos | 1600 | pos |
| 18 | NA | pos | pos | 1600 | neg |
| 19 | NA | pos | pos | 3200 | pos |

**Key:** pos, positive; neg, negative; NA, not available; PhIP-Seq, phage immunoprecipitation sequencing; CBA, cell-based assay; ELISA, enzyme-linked immunosorbent assay.

**Supplementary Table 2:** Summary of Healthy Control and Disease Control Patient Cohorts Tested by Cavin-4 IgG Peptide and Protein ELISA.

|  | **Peptide ELISA** | **Protein ELISA** |
| --- | --- | --- |
| iRMD | 19 | 19 |
| HC | 47 | 120 |
| AMA | 12 | 12 |
| ANA | 87 | 87 |
| HGG | 21 | 21 |
| IBM | 25 | 25 |
| ALS | 69 | 104 |
| MRS | 221 | 151 |
| MS | 26 | 8 |
| NAM | na | 191 |
| Suspected AE | na | 822 |
| PNH | 81 | 81 |

control cohort

**Key:** iRMD, immune-mediated rippling muscle disease; AMA, antimitochondrial antibody; ANA, antinuclear antibody; HGG, hypergammaglobulinemia; IBM, inclusion body myositis; ALS, amyotrophic lateral sclerosis; MRS, myositis related syndrome; MS, multiple sclerosis; NAM, necrotizing autoimmune myopathies; AE, autoimmune encephalitis; PNH, peripheral nerve hyperexcitability syndrome (isaac’s/cramp-fasciculation); na, not available.

**Supplementary Table 3:** Summary of individual patient discordant results including clinical presentation, genetic testing results, muscle pathology and response to immunotherapy.

| **Pt#** | **Clinical presentation** | **Caveolin 3 and Cavin-1 gene sequencing** | **Muscle biopsy** | **Immunotherapy** | **Post treatment clinical presentation** |
| --- | --- | --- | --- | --- | --- |
| P9 | Presented with myalgia and fatigue. Diffuse muscle rippling (sparing bulbar muscles), percussion-induced muscle rippling and muscle mounding. | Negative | mosaic pattern of cavin-4 and caveolin-3 immunostaining with a low percentage (18%) of muscle fibers showing attenuation of cavin-4 sarcolemmal immunoreactivity | Intravenous methylprednisolone, 500 mg weekly for 4 weeks, with maintenance of intravenous immunoglobulin, 0.4 g/kg, and azathioprine, 2 mg/kg, daily thereafter. | Remission |
| P10 | Presented with myalgia and fatigue & proximal weakness. Diffuse muscle rippling (sparing bulbar muscles), percussion-induced muscle rippling and muscle mounding were also seen. | Negative | normal caveolin-3 immunoreactivity; some chronic neurogenic changes and the lobulated the fibers. | Monthly intravenous immunoglobulin, 2g/kg | Stabilization |

**Supplementary Table 4:** PhIP-Seq enrichment scores in sera from patients with myositis related syndrome and interstitial lung disease with false-positive or borderline negative cavin-4 IgG results on peptide ELISA.

|  | **Mixed connective tissue disease** | **Anti synthetase syndrome** | **Mixed connective tissue disease** | **Polymyalgia Rheumatica** | **Anti synthetase syndrome** | **Inflammatory polyarthritis** |
| --- | --- | --- | --- | --- | --- | --- |
| NP_001018126.1\|240-289\|CAVIN4 | 0.33 | 1.0 | 0.33 | 0.67 | 1.67 | 1.0 |
| NP_001018126.1\|264-313\|CAVIN4 | 0.47 | 0.47 | 0.32 | 0.95 | 0.63 | 0.95 |
| NP_001018126.1\|288-337\|CAVIN4 | 2.19 | 6.23 | 0.92 | 0.46 | 0.92 | 1.27 |
| NP_001018126.1\|96-145\|CAVIN4 | 0.50 | 1.0 | 1.50 | 0.50 | 1.0 | 1.50 |
| NP_001018126.1\|144-193\|CAVIN4 | 0.27 | 0.55 | 0.27 | 0.27 | 0.82 | 0.55 |
| NP_001018126.1\|192-241\|CAVIN4 | 0.75 | 0.25 | 0.50 | 0.25 | 0.50 | 1.0  PhiP-Seq Enrichment score |
| NP_001018126.1\|312-361\|CAVIN4 | 2.0 | 2.0 | 1.00 | 1.0 | 2.0 | 2.0 |
| NP_001018126.1\|216-265\|CAVIN4 | 0.69 | 0.46 | 0.69 | 0.23 | 0.23 | 0.23 |
| NP_001018126.1\|CTERM\|CAVIN4 | 0.60 | 0.60 | 0.60 | 0.60 | 0.60 | 0.60 |
| NP_001018126.1\|120-169\|CAVIN4 | 0.75 | 0.75 | 0.75 | 0.75 | 0.75 | 0.75 |
| NP_001018126.1\|24-73\|CAVIN4 | 0.50 | 0.50 | 0.50 | 1.0 | 0.50 | 0.50 |
| NP_001018126.1\|48-97\|CAVIN4 | 1.0 | 1.0 | 1.0 | 1.0 | 1.0 | 1.0 |
| NP_001018126.1\|168-217\|CAVIN4 | 1.0 | 1.0 | 1.0 | 1.0 | 1.0 | 1.0 |
| Peptide ELISA OD | **1.15** | **2.28** | 0.88 | 0.87 | 0.99 | 0.75 |

oligonucleotide

**Key:** OD, optical density; ELISA, enzyme-linked immunosorbent assay; PhIP-Seq, phage immunoprecipitation sequencing. Bolded values yield ELISA cut-off scores of >1.0

**Supplementary Figure 1:** Comparison of Peptide and Protein ELISA ROC and Area Under the Curve in iRMD Patients as Compared to Healthy Controls. Optical density cut of at 1.0 provided 100% specificity, along with high clinical sensitivity.


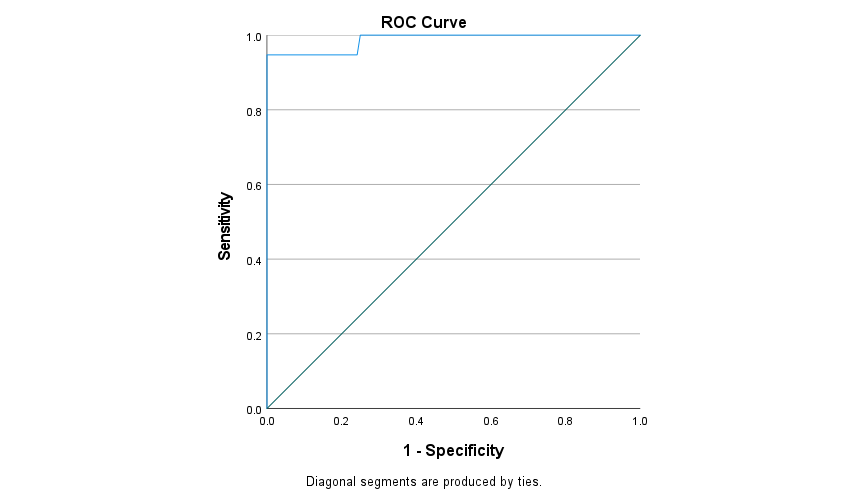

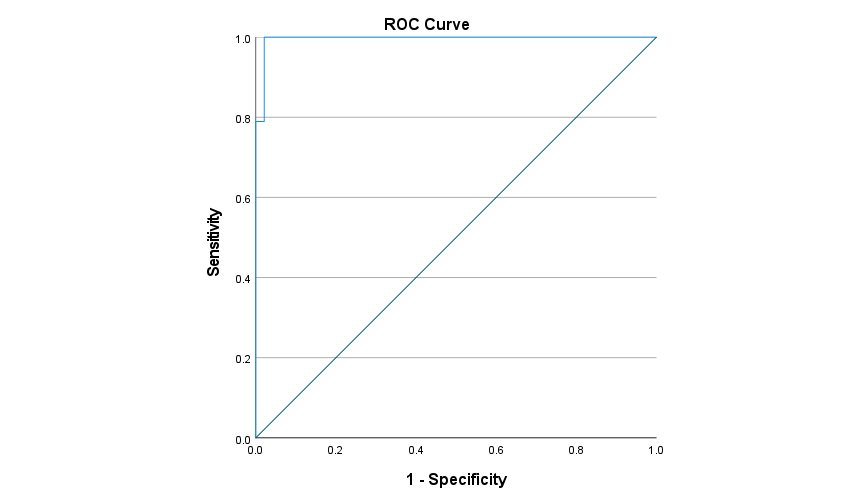


Peptide ELISA ROC

Protein ELISA ROC

Sensitivity

Sensitivity

Specificity

Specificity


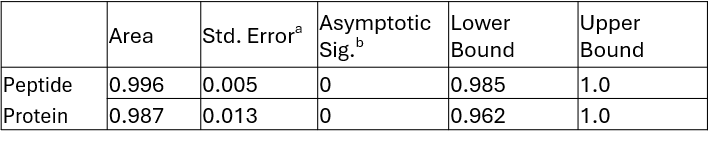


**Key:** a, under the nonparametric assumption; b, null hypothesis: true area = 0.5; ROC, receiver operating characteristic
